# Machine Learning-Powered fNIRS Detection of Idiopathic Central Precocious Puberty via Prefrontal Cortex Activation

**DOI:** 10.34133/bmef.0223

**Published:** 2026-03-25

**Authors:** Zeying Li, Lifang Jia, Yingxue Zou, Mengyu Jia, Limin Zhang, Dongyuan Liu, Feng Gao

**Affiliations:** ^1^ College of Precision Instruments and Optoelectronics Engineering, Tianjin University, Tianjin 300072, China.; ^2^ Academy of Medical Engineering and Translational Medicine, Tianjin University, Tianjin 300072, China.; ^3^ Tianjin Hospital of Tianjin, Tianjin 300211, China.; ^4^ Children’s Hospital of Tianjin University, Tianjin 300074, China.

## Abstract

**Objective and Impact Statement:** This study examines prefrontal cortex (PFC) hemodynamic responses in children with idiopathic central precocious puberty (ICPP) versus normals and constructs a noninvasive diagnostic model using functional near-infrared spectroscopy (fNIRS) augmented by machine learning. **Introduction:** Current ICPP diagnosis relies on invasive and time-consuming gonadotropin-releasing hormone stimulation tests. While fNIRS offers a noninvasive alternative, the neural mechanisms underlying ICPP remain unclear, and reliable automated diagnostic tools distinguishing patients from healthy peers are lacking. **Methods:** fNIRS data were acquired from 167 participants (82 ICPP and 85 normal) during a mental arithmetic (MA) task. General linear models and statistical tests were employed to analyze group and gender-specific activation patterns. Multidimensional features were extracted from hemodynamic signals, and a conditional denoising diffusion probabilistic model (C-DDPM) was introduced for data augmentation. **Results:** Analysis revealed gender-specific disparities, with the normal group exhibiting more extensive PFC activation than the ICPP group. In classification, a decision tree model using features from key negatively correlated channels achieved 86.57% accuracy. Notably, integrating C-DDPM-generated synthetic data further improved classifier performance metrics. **Conclusion:** The study elucidates the mechanisms of PFC activation in both normative and ICPP-affected cohorts during MA tasks and validates the effectiveness of machine learning in distinguishing between normal and ICPP children. This study provides a scientific basis for the development of automated, noninvasive rapid diagnostic tools for ICPP.

## Introduction

Precocious puberty is a prevalent endocrine ailment observed in prepubertal juveniles, demonstrating a mounting global prevalence. Recent investigations have unveiled intriguing evidence suggesting gender-specific variations in causative factors [1–4]. Precocious puberty is classified into 2 types: central precocious puberty and peripheral precocious puberty. Approximately 90% of girls and 25% to 60% of boys with precocious puberty are diagnosed with idiopathic central precocious puberty (ICPP), which results from the premature activation of the hypothalamic–pituitary–gonadal axis (HPGA). This condition is due to early central nervous system activity, which leads to increased hormone levels and accelerates the development of the reproductive organs [5,6]. The premature activation of pubertal processes can lead to early fusion of the epiphyseal plates, thereby compromising final adult height and increasing susceptibility to morbidities such as diabetes, cancer, and cardiovascular disorders, as well as psychological and social adjustment issues. These long-term health issues pose a serious threat to an individual’s quality of life. Consequently, the timely identification and implementation of diagnostic and therapeutic measures are crucial for both the children affected by ICPP and the well-being of their families [7]. Currently, clinical diagnosis of ICPP requires a comprehensive integration of sex hormone levels, assessment of skeletal maturity, and outcomes derived from gonadotropin-releasing hormone (GnRH) stimulation tests. Unfortunately, these methods require x-ray examinations and frequent blood draws, which not only are physically harmful and time-consuming but also impose heavy economic and psychological burdens on pediatric patients and their families. Therefore, given the potential long-term consequences, there is a critical need to allocate resources toward developing more efficient and noninvasive diagnostic tools for ICPP. Such advancements would not only alleviate the burden on the healthcare system but also improve the long-term health outcomes for a growing population of affected children.

The premature onset of the HPGA may precipitate a substantial discharge of GnRH, provoking heightened levels of luteinizing hormone (LH), follicle-stimulating hormone (FSH), estradiol, and testosterone consequently engendering the initiation of sexual maturation. Research has indicated that the development and maturation of the prefrontal cortex (PFC) are tightly regulated by puberty hormones [8]. Notably, excessive exposure to high levels of sex hormones during critical stages of brain development can lead to permanent changes in brain structure and function [9–12]. Chen et al., through resting-state magnetic resonance imaging (MRI) and functional magnetic resonance imaging (fMRI), found that girls with ICPP exhibited reduced gray matter volume in the left insula and left fusiform gyrus. Additionally, connectivity between the left and right insula and the right middle frontal gyrus, as well as between the left fusiform gyrus and right amygdala, was reduced in these girls [13]. Previous neuroimaging studies have shown that familial male precocious puberty is linked to structural and functional abnormalities in the brain, which are associated with cognitive impairment [14,15]. Research has demonstrated that sex hormones can directly or indirectly influence the development of the PFC. Estradiol can indirectly or directly influence the functional activity of the PFC by regulating the frontoparietal network, anterior insula, and anterior cingulate cortex, thereby playing a role in higher-order cognitive processes [16–18]. LH indirectly affects PFC function by modulating the cingulum and associated networks, such as the frontoparietal network [19]. FSH concentration is directly related to the volume of core prefrontal regions, including the left anterior cingulate cortex and left frontal areas, thereby affecting the development and function of the PFC [20]. Higher testosterone levels can reduce PFC thickness, thus affecting executive and cognitive functions [21]. These discoveries facilitate a physiological foundation for employing advanced brain imaging technology as a means of screening for ICPP [22,23]. Indeed, imaging investigations pertaining to ICPP predominantly center around MRI and fMRI [24,25]. While the pathological origin of ICPP lies in the premature activation of the deep-brain HPGA, these MRI/fMRI findings demonstrate that the resulting hormonal cascade has measurable downstream consequences on cortical structure and function. However, although these findings are crucial for understanding the neural underpinnings of ICPP, their role as stand-alone diagnostic tools in clinical practice remains limited. The current clinical diagnosis still relies on invasive and time-consuming methods like GnRH stimulation tests. Moreover, the limitations of fMRI, such as its high cost, high noise levels, and the requirement for subjects to remain motionless, render it unsuitable for routine screening. This gap highlights the urgent need for accessible, noninvasive methods like functional near-infrared spectroscopy (fNIRS) that can capture the cortical functional changes associated with ICPP.

fMRI is used to directly detect changes in blood flow to active areas of the brain, while fNIRS indirectly assesses brain activity by monitoring changes in blood oxygen levels within brain tissue. The detection of blood flow changes in brain activity areas and the monitoring of blood oxygen levels in brain tissue are closely related. This relationship is primarily manifested through the neurovascular coupling mechanism, which describes the interaction between neural activity and blood flow dynamics in the brain. Structural and functional abnormalities in the PFC of children with ICPP result in task-induced neurovascular coupling mechanisms that differ from those of normal children, and these differences can be detected using fNIRS. However, there is currently no research focusing on the cerebral functional attributes of individuals with ICPP utilizing fNIRS in the contemporary scientific landscape.

fNIRS is an optical imaging technique that has now been demonstrated to be an effective tool for investigating brain physiological states by detecting concentrations of oxy-hemoglobin (HbO) and deoxy-hemoglobin (HbR) [26–30]. It offers advantages such as environmental robustness, nonradioactivity, noninvasiveness, noiselessness, and portability, and is increasingly applied in brain research [31,32]. Mental arithmetic (MA), which requires short-term memory and cognitive strategies, is closely related to PFC activity [33–35]. This study employed fNIRS, a brain imaging tool, to investigate the activation patterns in the PFC region of 167 adolescent participants during the MA task. These patterns were subsequently used to develop and evaluate refined classification models for clinical screening of ICPP. To further investigate the impact of an increased dataset size on classification performance, we employed a conditional denoising diffusion probabilistic model (C-DDPM) to generate additional synthetic data.

## Results

**Fig. 1.**
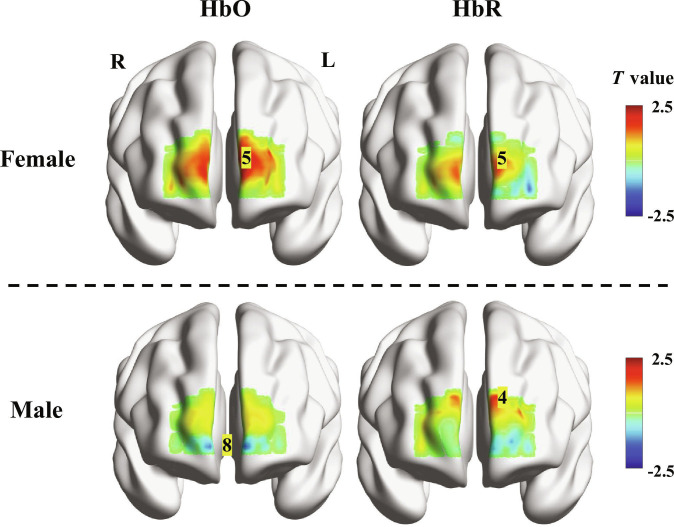
Two-sample *t* test chart between normal and ICPP groups of females (top) and males (bottom) during the MA period. The channels marked with numbers represent channels with significant differences (*P* < 0.05).

**Fig. 2.**
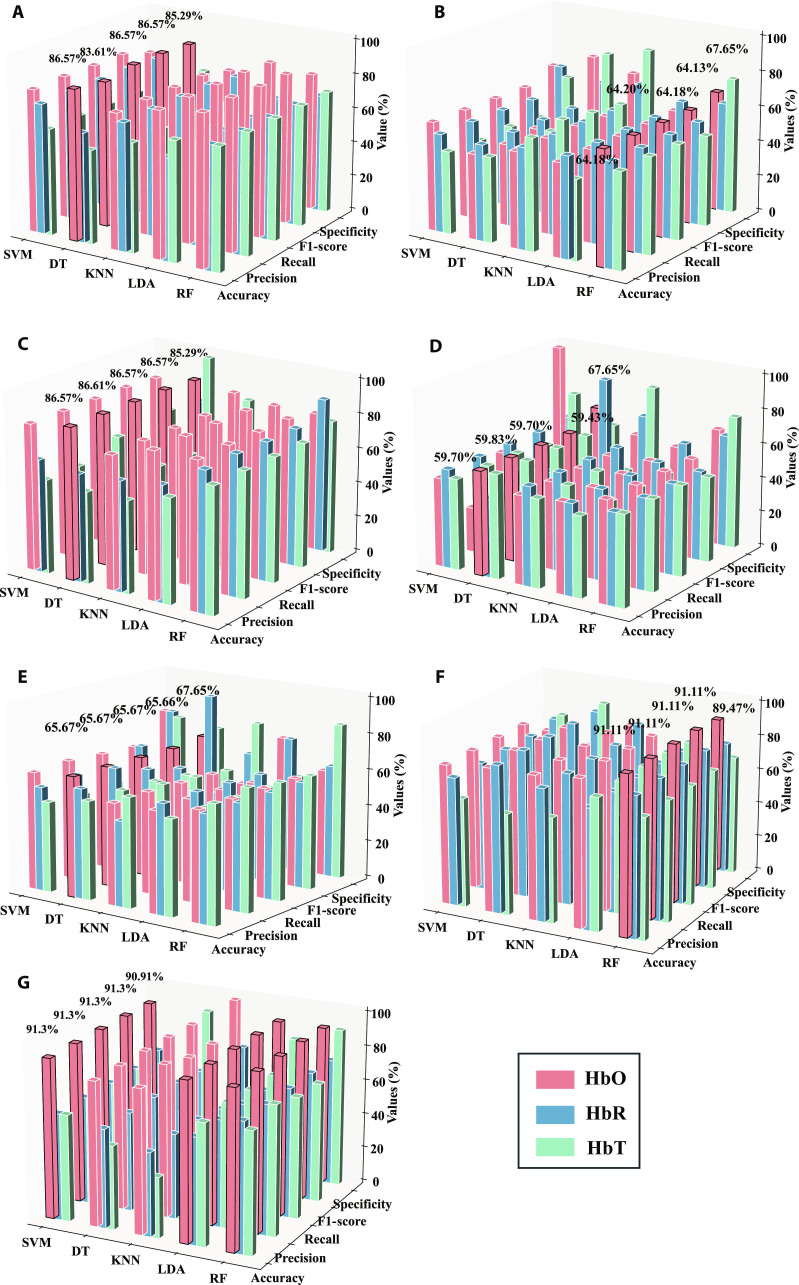
Classification performance using various hemoglobin features. This figure presents results from the test set, illustrating the impact of different hemoglobin features on classification accuracy. (A) to (E) display results without gender consideration, while (F) and (G) show results exclusively for females and males, respectively. Feature descriptions: (A) Feature set A. (B) Feature set B. (C, F, and G) Feature set C. (D) Feature set D. (E) Feature set E.

In this study, we analyzed fNIRS signals by gender to identify differences in PFC activation between the normal and ICPP group during the MA task. The results are presented in Tables 1 and 2 and Fig. 1. Additionally, 5 different types of features were extracted from ΔHbO, ΔHbR, and total hemoglobin changes (ΔHbT) and input into support vector machines (SVM), random forest (RF), decision tree (DT), linear discriminant analysis (LDA), and k-nearest neighbors (KNN) classifiers. After optimizing the model hyperparameters, the best classification results for each feature are shown in Fig. 2. The specific results are as follows.

**Table 1.** Paired sample *t* test comparing females in the normal and ICPP groups during the MA versus rest periods. Only significant results were reported in the table.

Hemoglobin	Channels	*T* value	Adjusted *P* value	Channels	*T* value	Adjusted *P* value
	Normal			ICPP		
HbO	3	2.436	0.018	– ^a^	– ^a^	– ^a^
HbR	4	−2.414	0.019	3	−2.977	0.004
	6	−2.624	0.011	4	−3.401	0.001
	9	−2.016	0.048	10	−2.255	0.028
HbT	2	2.418	0.019	1	2.837	0.006
	3	4.595	0.000	3	4.077	0.000
	4	4.350	0.000	4	4.344	0.000
	6	3.069	0.003	7	2.324	0.024
	7	2.295	0.026	8	2.818	0.007
	8	2.333	0.023			
	9	2.181	0.033			
	10	3.270	0.002			

^a^ No significant result in this hemoglobin.

**Table 2.** Paired sample *t* test comparing males in the normal and ICPP groups during the MA versus rest periods. Only significant results were reported in the table.

Hemoglobin	Channels	*T* value	Adjusted *P* value	Channels	*T* value	Adjusted *P* value
	Normal			ICPP		
HbO	4	2.546	0.017	8	4.010	0.000
	5	2.236	0.034			
HbR	– ^a^	– ^a^	– ^a^	3	−4.513	0.000
				4	−2.863	0.008
HbT	3	2.327	0.028	1	3.671	0.001
	4	2.262	0.032	3	5.803	0.000
	10	2.581	0.016	4	3.913	0.001
				8	2.308	0.029

^a^ No significant result in this hemoglobin.

### Brain activation level

A paired sample *t* test was conducted to evaluate brain activation levels by comparing the *β* values during the MA and rest periods, with only statistically significant results reported in Table 1 (females) and Table 2 (males).

In females, significant activation for HbO was observed in channel 3 in the normal group, but not in the ICPP group. For HbR, significant activation was observed in channels 4, 6, and 9 in the normal group, while channels 3, 4, and 10 showed significant activation in the ICPP group. For HbT, all channels except 1 and 5 exhibited significant activation in the normal group, compared to significant activation in channels 1, 3, 4, 7, and 8 in the ICPP group. These findings suggest that the normal group displays more extensive activation across the PFC during the MA period compared to the ICPP group.

Regarding the male participants, significant activation for HbO was found in channels 4 and 5 in the normal group, and channel 8 in the ICPP group. For HbR, significant activation was observed in channels 3 and 4 in the ICPP group, with no corresponding activation in the normal group. For HbT, channels 3, 4, and 10 were significantly activated in the normal group, while channels 1, 3, 4, and 8 showed significant activation in the ICPP group. These analyses indicate that females exhibit a higher number of activated channels compared to males during the MA period. Additionally, the *t* values indicate that the ICPP groups exhibited stronger negative activation in HbR compared to their normal counterparts.

### Differences in brain activation

A 2-sample *t* test was conducted to evaluate brain activation differences during the MA period, as depicted in Fig. 1, where channels with significant differences are numbered. In males, channel 8 for HbO demonstrated significant differences in activation, showing lower activation compared to the ICPP group (*t* = −2.582, *P* = 0.013). Channel 4 for HbR also showed significant differences, with higher activation in the normal group compared to the ICPP group (*t* = 2.100, *P* = 0.040). In females, channel 5 for both HbO and HbR displayed significant differences in activation during the MA task, with higher activation in the normal group compared to the ICPP group (*t* for HbO = 2.507, *P* for HbO = 0.014; *t* for HbR = 2.056, *P* for HbR = 0.042).

These findings highlight distinct activation patterns between the normal and ICPP groups across both genders during the MA task, emphasizing the importance of considering both gender and pathological status when assessing the neural underpinnings of precocious puberty. The normal group typically exhibited stronger brain activation, suggesting that premature exposure to excessively high levels of sex hormones may affect the development of the PFC.

### Clinical data-driven classification

Figure 2 displays the classification performance of the results. The goal is to explore the data features and identify the optimal disease classification model. Only the best model for each feature is marked in the figure. Detailed classification model results for each feature are shown in Tables S1, S2, and S3.

**Fig. 3.**
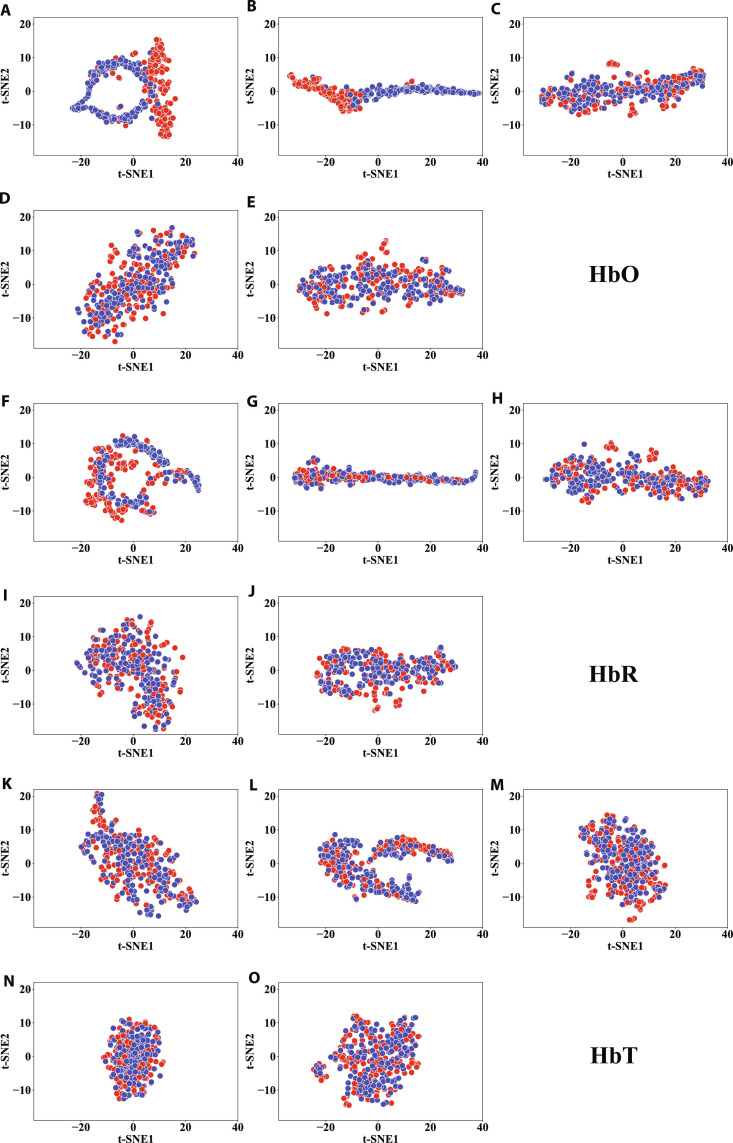
Visualization of different features in 2D using t-SNE. The subplots represent feature sets for HbO (A to E), HbR (F to J), and HbT (K to O). Within each hemoglobin species group, the panels correspond to the following feature sets: A/F/K: Feature set C; B/G/L: Feature set A; C/H/M: Feature set B; D/I/N: Feature set D; and E/J/O: Feature set E. Color: blue for normal group and red for ICPP group.

**Fig. 4.**
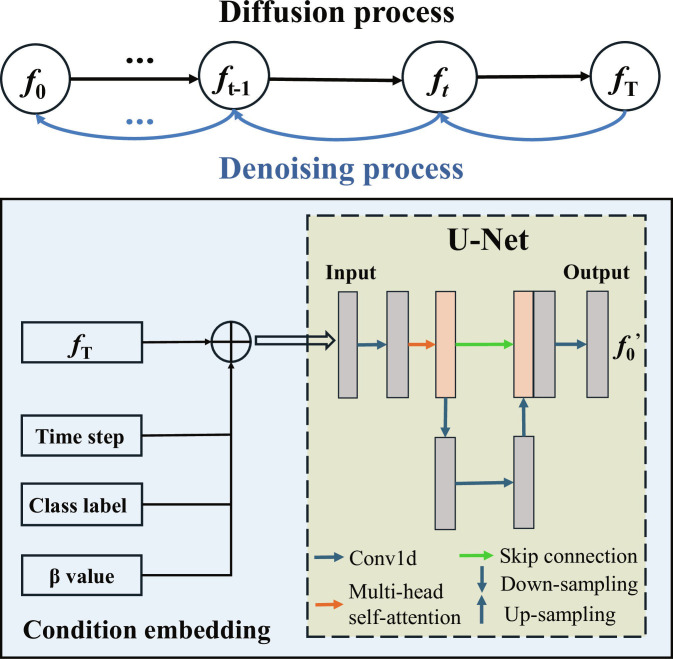
Forward and denoising steps in the C-DDPM model. Here, *f*_0_ represents the preprocessed fNIRS data, *f*_T_ denotes the fNIRS data that has undergone forward diffusion and been added noise through T steps, and *f*_0_′ is the generated fNIRS data.

Firstly, classification models were developed based on the normal and ICPP groups without considering gender, yielding favorable results as shown in Figs. 2A to 4E. In a preliminary comparison of classification results from the 3 hemoglobin concentration signals (HbO, HbR, and HbT), the HbO signal demonstrated optimal classification performance. For the HbO signal, Features B, D, and E achieved accuracy rates of 64.18%, 59.70%, and 65.67%, respectively, under the optimal model. In contrast, using Feature A alone led to a substantial enhancement in model performance. Its accuracy reached 86.57%, with a precision, recall, F1 score, and specificity of 83.61%, 86.57%, 86.57%, and 85.29%, respectively. In pursuit of further performance gains, a composite feature set (Feature C) was constructed. This set combines the time-domain characteristics of the strongest and most frequently occurring negatively correlated channels with the first 3 principal components extracted from corresponding channel using principal component analysis (PCA). The optimal model based on Feature C achieved the best overall performance in this study: while its accuracy, recall, F1 score, and specificity were on par with those of Feature A, its precision further increases to 86.61% (see Fig. 2C). Compared to Feature A, Feature C achieved a 3-percentage-point increase in precision. This improvement indicates that the composite features, while maintaining a high patient identification rate, are more effective at reducing the probability of misclassifying normal individuals as patients.

Additionally, we constructed separate classification models for the normal and ICPP groups for each gender. The best results are shown in Fig. 2F and G; the results showed that the best feature is still Feature A or Feature C. Among males, Features A and C of HbO both yielded the best classification performance, with an accuracy of 91.30%, a precision of 91.30%, a recall of 91.30%, an F1 score of 91.30%, and a specificity of 90.91%. Among females, inputting Feature C of HbO into RF resulted in the best classification performance, with an accuracy of 91.11%, a precision of 91.11%, a recall of 91.11%, an F1 score of 91.11%, and a specificity of 89.47%. Detail classification model results of each feature by males and females are shown in Tables S2 and S3. These results effectively distinguish the ICPP group from the normal group.

Furthermore, we employed the t-distributed stochastic neighbor embedding (t-SNE) method to visualize the 5 features extracted from the 3 types of hemoglobin, respectively, as shown in Fig. 3. The t-SNE method, a widely used visualization technique, reduces high-dimensional data into a 2-dimensional space [36,37], enabling a clearer analysis of the feature distributions and patterns across the different types of hemoglobin. From Fig. 3A and B, it can be intuitively seen that Features A and C of HbO can better distinguish between normal and ICPP groups.

## Discussion

### Gender-specific effects of ICPP on PFC activation during MA tasks

In this study, we utilized fNIRS technology to investigate the PFC brain activation characteristics during MA tasks in the normal group and ICPP group, and analyzed the influence of gender on this process. The main findings show that normal females exhibit more activation channels in HbO and HbT compared to ICPP females, and there are significant differences in the activation patterns of HbO and HbR between the normal and ICPP groups, regardless of gender. These results indicate that premature activation of the HPGA alters the function of the PFC, thereby altering neurophysiological mechanisms.

The normal female group showed more extensive PFC activation in HbO and HbT, which may reflect the maturation of PFC function and task-related cognitive load processing ability during normal sexual development. In contrast, females with ICPP showed reduced activation in the left PFC region, indicating that early and excessive exposure to sex hormones may interfere with the normal development and functional integration of the left PFC in females. This finding is consistent with previous research [13]. In terms of males, there are differences in the activation areas between the normal group and ICPP group, especially in the intra-abdominal PFC and left dorsolateral PFC. These regions are closely related to emotional regulation, decision-making, and executive functions [38]. The abnormal activation of these regions in ICPP females and males may reflect the interference of excessive sex hormones on these cognitive processes. These findings are consistent with previous studies on the critical role of sex hormones in brain development, where premature activation of HPGA not only leads to PFC dysfunction but may also increase the risk of developing Alzheimer’s disease [39]. This result supplements the understanding of gender differences in brain function under the influence of ICPP, emphasizing the unique role of sex hormones in female and male brain development.

In addition, the activation differences between male and female groups indicate that the impact of ICPP on brain function is gender specific. This may be related to the levels of sex hormones, receptor expression, and their distribution in different regions of the brain in men and women. This discovery helps explain why ICPP may exhibit different clinical symptoms and behavioral characteristics in different genders [40].

### Relationships between brain activation and demographic and clinical data

To further investigate the biological underpinnings of our findings, we performed Pearson correlation analyses to explore the relationship between brain activation (*β* values in channels with significant group differences) and demographic/clinical data. The detailed results of these analyses are presented in Tables S4 and S5.

Our analysis revealed no significant correlation between brain activation and demographic variables such as age or education in any group (*P* > 0.05). Within the ICPP group, we investigated the relationship between clinical markers and brain activation. A significant positive correlation was found between FSH levels and HbO activation in channel 5 for girls with ICPP (*r* = 0.360, *P* = 0.029). This indicates that higher FSH levels were associated with stronger cortical activation in this region. No significant correlations were observed between any clinical markers and brain activation in boys (*P* > 0.05).

A key finding from our analysis is the significant positive correlation between FSH levels and PFC activation, found exclusively in girls with ICPP. This provides evidence directly linking a specific hormonal marker to the observed alterations in cortical function. This finding reinforces the theme of gender-specific neural effects of ICPP, suggesting that FSH may play a unique role in modulating PFC activity during cognitive tasks in the female population. No significant correlations were found in boys, despite some trends (e.g., a nonsignificant negative correlation between FSH and HbR activation, *P* = 0.053). Additionally, the lack of correlation with age across all groups strengthens our main finding that the observed differences in brain activation are primarily driven by the pathological condition rather than developmental stage alone. Our findings establish a brain–hormone relationship that strengthens the biological plausibility of using fNIRS to detect ICPP-related neural changes.

### Superiority of negatively correlated HbO features for ICPP classification

The classification results indicate that, regardless of whether gender is taken into account, the best performance was achieved using features obtained by Feature A or Feature C in the HbO signals. In contrast, the other 3 features of HbO, as well as those extracted from HbR and HbT, did not produce comparable results. Research has shown that children with ICPP experience changes in brain functional connectivity [13]. In the study of functional connectivity between positive and negative networks, extracting features from the negative network and inputting them into the model resulted in superior classification performance compared to the positive network [41]. This can further explain why the highest classification accuracy was achieved in this study by inputting the features of the channels with the most negative correlations. By selecting the most negatively correlated channel pairs, we focus on brain regions that may reflect significant functional differences. This indicates that the activity of the 2 brain regions is inversely synchronized, with an increase in activity in one region and a decrease in activity in the other. The results of this study also indicate that there are differences in this phenomenon between normal children and children with ICPP. Time-domain features (mean, variance, skewness, and kurtosis) extracted from these 2 channels provide a comprehensive description of signal dynamics [42], while PCA is utilized to capture primary variation patterns [43]. Combining these 2 types of features into the optimized model can effectively distinguish between normal children and ICPP children. In this study, the best classification performance was achieved using features extracted from HbO signals. This is because HbO signals have a higher signal-to-noise ratio and greater sensitivity to neural activity compared to HbR and HbT signals [44]. Consequently, many studies choose to analyze only HbO signals [45,46]. Although the other 3 feature extraction methods did not yield optimal results in this study, they still provide new ideas for feature extraction of fNIRS data. These findings highlight the potential of machine learning in using fNIRS to enhance ICPP diagnostic accuracy, offering a promising avenue for the development of automated and noninvasive diagnostic tools.

### The potential of C-DDPM to improve classification performance

The limited amount of data may be a potential issue affecting the classification results of this study. Therefore, we investigated whether a larger dataset could further improve accuracy. Based on this, we attempted to generate more data using a generative model. While generative approaches like conditional generative adversarial networks have become a tool for fNIRS data synthesis [47], they are often plagued by challenges such as training instability and mode collapse. More recently, since the introduction in 2015 [48] and popularization by Ho et al. in 2020 [49], denoising diffusion probabilistic model has shown exceptional performance in image generation tasks and has begun to be applied to data augmentation in fNIRS data [50]. In order to ensure that the generative model can learn the brain activation patterns of the normal group and the ICPP group, we introduced C-DDPM (see Fig. 4). The framework operates through a 2-stage process: during the forward diffusion process, Gaussian noise is progressively added to the original fNIRS signal data until it transforms into pure random noise; subsequently, a U-Net neural network featuring multihead self-attention mechanisms, up-sampling and down-sampling layers, and skip connections is trained to learn the reverse denoising process. To ensure the generation of class-specific data, we conditioned the reverse process not only on the timestep but also on class labels and *β* values. This guidance enables the network to reconstruct high-fidelity fNIRS signals unique to each group. The C-DDPM network was trained using a mean squared error loss function. The model is configured with a linear noise schedule ranging from 0.001 to 0.02 across 200 diffusion timesteps, trained for 500 epochs with a batch size of 64, and optimized using the AdamW optimizer with a learning rate of 0.001 and a weight decay of 0.01. The programming was done using Python 3.11.7 and PyTorch 2.5.1, with experiments conducted on an Nvidia GeForce RTX 4070 GPU.

We used C-DDPM to generate HbO data equal in size to the original training set and included it in the training samples, while keeping the test set unchanged and composed entirely of real data. We then extracted the best features (Features A and C) and retrained our classification model on this augmented dataset, before evaluating their performance on the real test dataset.

The classification results with the addition of synthetic data are shown in Table 3, where bold values indicate improved performance metrics. A clear and consistent trend can be seen from the comparison: the application of data augmentation based on C-DDPM markedly improved the specificity metrics of almost all models. Furthermore, after data augmentation, all reported metrics of each model for feature A exceeded 80%, and all reported metrics of each model for feature C exceeded 83%, establishing a new high-robustness baseline. These findings collectively validate the efficacy of our C-DDPM augmentation strategy in enhancing the robustness and diagnostic accuracy of fNIRS-based classification. In the future, we will continue to collect more clinical data and use data generation methods to generate more clinical data to improve the practicality of our work.

**Table 3.** Comparative performance of machine learning classifiers on original versus C-DDPM-augmented HbO data. Bold values indicate improved performance metrics.

Data set	Feature set	Algorithm	Accuracy	Precision	Recall	F1 score	Specificity
		LDA	83.58	83.62	83.58	83.58	82.35
		SVM	82.09	82.58	82.09	82.04	76.47
	Feature A	DT	86.57	83.61	86.57	86.57	85.29
		RF	86.57	87.43	86.57	86.51	79.41
		KNN	77.61	77.86	77.61	77.58	73.53
Real							
		LDA	83.58	83.87	83.58	83.56	79.41
		SVM	83.58	83.62	83.58	83.58	82.35
	Feature C	DT	86.57	86.61	86.57	86.57	85.29
		RF	83.58	83.87	83.58	83.56	79.41
		KNN	76.12	76.46	76.12	76.01	82.35
		LDA	83.58	83.60	83.58	83.57	**85.29**
		SVM	**83.58**	**83.03**	**83.58**	**83.54**	**88.24**
	Feature A	DT	86.57	**86.05**	86.57	86.53	**91.18**
		RF	85.07	85.07	85.07	85.07	**85.29**
		KNN	**80.60**	**80.82**	**80.60**	**80.54**	**85.29**
Real + Synthetic							
		LDA	83.58	83.60	83.58	**83.57**	**85.29**
		SVM	83.58	85.07	83.58	83.37	**94.12**
	Feature C	DT	86.57	86.61	86.57	86.57	85.29
		RF	83.58	83.60	83.58	**83.57**	**85.29**
		KNN	**83.58**	**83.60**	**83.58**	**83.57**	**85.29**

### Limitations and outlook

This study has certain limitations. First, regarding data acquisition and processing, the study was conducted at a relatively low sampling rate and only used low-pass filtering to remove physiological noise. In the future, employing equipment with a higher sampling rate, combined with short-range measurements, could be considered. This will not only help to more accurately characterize and remove physiological noise on the scalp surface, but will also ultimately improve overall data quality and signal fidelity. In future work, we will continue to collect clinical data. In addition, we will optimize the C-DDPM model and develop a deep learning-based classifier to achieve end-to-end, fully automated classification of ICPP, thereby providing an efficient and accurate auxiliary tool for clinical diagnosis.

The integration of fNIRS and optical imaging technologies into the study of ICPP holds great promise for advancing both clinical and research applications. In the future, fNIRS can be applied to the early detection of ICPP-related neural changes, thereby helping doctors to achieve timely intervention and treatment of ICPP and alleviate the suffering of patients and their families. Additionally, optical imaging can provide noninvasive, real-time monitoring of brain activity changes in response to therapeutic treatments, thereby enhancing the ability to assess treatment efficacy and personalize therapeutic strategies. Furthermore, these technologies offer the potential to elucidate the underlying neural mechanisms driving premature pubertal development, contributing to a more comprehensive understanding of ICPP. With the continuous development of fNIRS and optical imaging technology capabilities, their application in longitudinal studies can reveal the developmental trajectory of affected children, ultimately providing information for developing targeted intervention measures and improving long-term prognosis.

## Conclusion

In this study, we used fNIRS technology for the first time to explore the differences in brain function between normal children and ICPP patients. The main purpose of the study was to evaluate the feasibility of fNIRS as a diagnostic tool for ICPP and its unique advantages, especially as a noninvasive, rapid, and easy-to-implement screening method. By comparing the activation patterns of the PFC between ICPP groups and normal groups in MA tasks, we not only revealed the potential differences in brain functional activities between the 2 groups, but also evaluated the effectiveness of different machine learning algorithms in identifying ICPP groups.

Our results provide important insights for the timely identification and therapeutic intervention of ICPP, and demonstrate the broad prospects of fNIRS in future clinical applications. With the continuous advancement of technology, fNIRS is expected to become a key tool for the diagnostic evaluation and longitudinal monitoring of ICPP and other neuroendocrine diseases.

## Materials and Methods

### Participants

An a priori power analysis was conducted using G*Power 3.1 to determine the necessary sample size [51]. Our primary analysis involved comparing the differences in PFC activation between the normal group and the ICPP group using an independent-samples *t* test. This calculation was based on a statistical power (1 − *β*) = 0.80, an error probability of *α* = 0.05, and an effect size of *d* = 0.5. The formula used for this estimation is as follows:

$$n=\frac{2\times {\left(Z_{\alpha /2}+Z_{\beta }\right)}^{2}}{d^{2}}$$ (1)

where $Z_{\alpha /2}$ is the standard normal deviate for *α*, $Z_{\beta }$ is the standard normal deviate for *β*, and *d* is the effect size. Based on these parameters, the analysis indicated that a minimum of 64 participants per group, for a total of 128 participants, was required. Participant details are presented in Table 4. Data were collected from 167 juvenile participants at Tianjin Hospital during April 2023 to July 2024, including 85 children with normal development and 82 children diagnosed with ICPP. The sample included 56 males (age, 11.8 ± 2.5 years) and 111 females (age, 10.3 ± 2.2 years), among whom 28 males and 54 females were diagnosed with ICPP. Regarding the patient distribution, there were significantly more female patients than male patients. The diagnostic criteria for ICPP included a bone age exceeding the corresponding chronological age by at least 1 year, premature onset of secondary sexual characteristics, and an LH peak greater than 3 IU/l during the GnRH stimulation test and a peak LH/FSH ratio ≥0.6 [52]. Diagnosis in females focuses primarily on estradiol levels, while in males, it focuses on testosterone levels. All procedures were conducted with prior approval from the ethics committees by the medical ethics review (MER) of Tianjin Hospital (2022MER130), and informed consent was obtained from the participants’ guardians.

**Table 4.** Demographic information for the normal and ICPP groups

Characteristics	Normal (*n* = 85)	ICPP (*n* = 82)	*t*/*χ*^2^	*P* value
Gender (female/male)	57/28	54/28	0.034	0.853
Education (years)	4.71 ± 2.48	4.91 ± 2.32	−0.548	0.585
Age (years)	10.67 ± 2.51	10.90 ± 2.37	−0.566	0.572
Bone age (years)	– ^a^	12.27 ± 1.88	– ^a^	– ^a^
LH (IU/l)	– ^a^	4.81 ± 3.47	– ^a^	– ^a^
FSH (IU/l)	– ^a^	4.76 ± 2.30	– ^a^	– ^a^
LH/FSH	– ^a^	0.99 ± 0.48	– ^a^	– ^a^
Estradiol (pmol/l)	– ^a^	118.58 ± 98.32 (female)	– ^a^	– ^a^
Testosterone (nmol/l)	– ^a^	12.75 ± 4.69 (male)	– ^a^	– ^a^

^a^ Only abnormal cases will have relevant diagnostic indicators archived.

### Instruments and experimental paradigm design

**Fig. 5.**
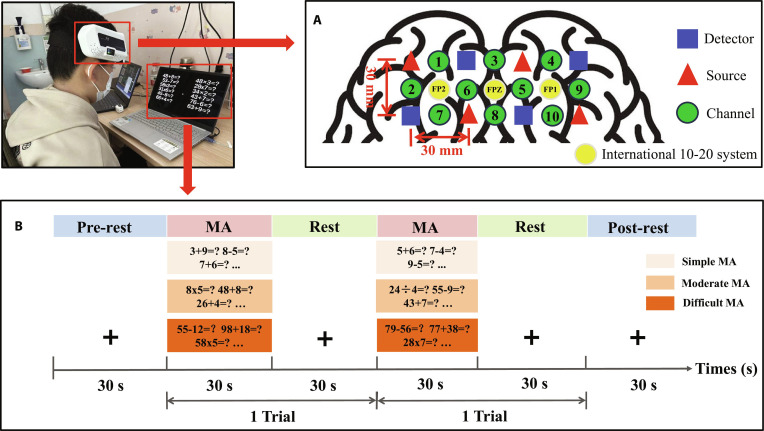
Experimental source, detector configuration (A) and experimental paradigm of MA (B).

This study utilized a portable and wearable continuous wave fNIRS device, the Brain-Explorer-Wear (PD-LED-10S2W4D26C), developed through a collaboration between NIRS Ergonomics (Xiamen) Technology Co., Ltd. and Tianjin University to collect data. The device employs a digital lock-in detection method, which allows simultaneous measurements from all channels at 2 distinct wavelengths (775 and 855 nm) [53]. It follows the 10-20 system layout and is equipped with 4 pairs of laser diode sources and 4 monolithic silicon photodiode detectors, arranged in a single lattice configuration. It can collect raw light intensity data from 20 channels (with each pair of light sources providing 10 sampling points at 2 wavelengths). The distance between each source and its corresponding detector is maintained at 30 mm, and the system operates at a data acquisition rate of 1 Hz. To ensure consistency across participants, the center of the probe was aligned with the Fpz landmark of the International 10-20 system. The layout of the sources and detectors according to this system is illustrated in Fig. 5A. The MNI coordinates for each channel were estimated by projecting this standardized probe layout onto an MNI standard brain template.

To ensure that children of different ages could successfully complete the experiment, we developed an MA paradigm with 3 distinct difficulty levels. Before the experiment, participants were asked to provide their age and education level to determine the appropriate difficulty level of the experimental paradigm. The specific details are as follows: for children under 8 years old, the MA tasks mainly involve single-digit addition and subtraction, with occasional double-digit operations. Children aged 9 to 12 are required to complete mostly double-digit addition and subtraction, along with a few instances of single-digit multiplication. Children over 13 years old are expected to focus primarily on double-digit addition and subtraction, with occasional double-digit multiplication. Participants were instructed to sit in a comfortable chair and minimize head movements after becoming familiar with the experimental requirements, prior to beginning the experiment. As illustrated in Fig. 5B, the paradigm consisted of alternating rest and MA phases. There are a total of 2 trials included, one of which includes a mental calculation phase and a rest phase. Before both the MA and rest tasks, a 2-s task prompt was presented, such as “Please rest for 30 seconds” or “Please perform mental arithmetic”. Following the prompt, participants immediately began the corresponding rest or MA task. During the rest phase, subjects should sit quietly and relax. During the MA phase, 10 arithmetic problems were displayed simultaneously on a screen, and participants were required to solve as many addition, subtraction, multiplication, and division problems as possible. Each phase lasted 30 s.

### fNIRS data preprocessing

It is an important step to conduct data quality assessment before data processing. Therefore, the coefficient of variation (*CV*) of the intensity data is calculated across all channels. The *CV* can be defined as:

$$\mathrm{CV}=\frac{\sigma \left(I\right)}{\mu \left(I\right)}\times 100\%$$ (2)

where I denotes the raw light intensity data, and $\sigma \left(\cdot \right)$ and $\mu \left(\cdot \right)$ denote the standard deviation and mean, respectively. Channels with *CV* exceeding 15% are classified as “bad”, and the bad-channel-related subject data are discarded. After that, the original light intensity data are converted into relative hemoglobin concentration using the modified Beer–Lambert law (MBLL) [54]. The specific formula is as follows:

$$\begin{array}{c} \mathrm{\Delta HbO}=\frac{\varepsilon_{\mathrm{HbR}}^{\lambda 2}\times \frac{\text{log}\left(I_{\lambda 1}^{0}/I_{\lambda 1}\right)}{{\mathrm{DPF}}_{\lambda 1}}-\varepsilon_{\mathrm{HbR}}^{\lambda 1}\times \frac{\text{log}\left(I_{\lambda 2}^{0}/I_{\lambda 2}\right)}{{\mathrm{DPF}}_{\lambda 2}}}{d\times \left(\varepsilon_{\mathrm{HbR}}^{\lambda 2}\times \varepsilon_{\mathrm{HbO}}^{\lambda 1}-\varepsilon_{\mathrm{HbR}}^{\lambda 1}\times \varepsilon_{\mathrm{HbO}}^{\lambda 2}\right)} \end{array}$$ (3)

$$\begin{array}{c} \mathrm{\Delta HbR}=\frac{\varepsilon_{\mathrm{HbO}}^{\lambda 2}\times \frac{\text{log}\left(I_{\lambda 1}^{0}/I_{\lambda 1}\right)}{{\mathrm{DPF}}_{\lambda 1}}-\varepsilon_{\mathrm{HbO}}^{\lambda 1}\times \frac{\text{log}\left(I_{\lambda 2}^{0}/I_{\lambda 2}\right)}{{\mathrm{DPF}}_{\lambda 2}}}{d\times \left(\varepsilon_{\mathrm{HbR}}^{\lambda 1}\times \varepsilon_{\mathrm{HbO}}^{\lambda 2}-\varepsilon_{\mathrm{HbR}}^{\lambda 2}\times \varepsilon_{\mathrm{HbO}}^{\lambda 1}\right)} \end{array}$$ (4)

**Fig. 6.**
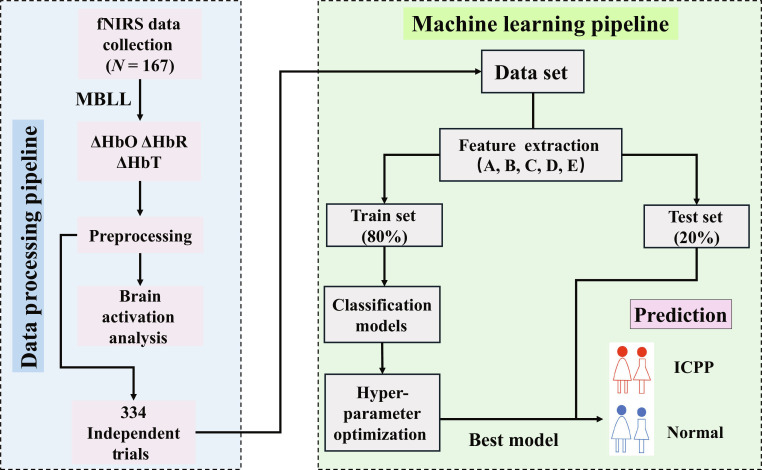
Flowchart of data preprocessing and machine learning pipeline.

ΔHbT=ΔHbO+ΔHbR (5)

where λ denotes the wavelength, $I^{0}$ represents the original intensity of light incident from the light source onto the tissue, I is the light intensity detected by the detector, d is the distance between the light source and the detector, DPF is the differential path length factor, and ε is the molar extinction coefficient. This study focuses on changes in ΔHbO, ΔHbR, and ΔHbT. The pipeline of preprocessing on fNIRS data is shown in Fig. 6.

In addition to task-induced brain activation signals, original signals also include physiological noise from heartbeat, breathing, and blood flow, as well as nonphysiological noise such as movement-related artifacts and equipment noise. Therefore, reliable signal preprocessing is crucial for accurately interpreting task-related brain activation. We utilized a polynomial regression model to estimate and remove the linear drift from the raw hemoglobin concentration signals. After that, a third-order Butterworth infinite impulse response (IIR) filter was applied with a low-pass cutoff at 0.1 Hz to eliminate physiological noise, such as heartbeat (>0.5 Hz), breathing (>0.2 Hz), Mayer wave (≈0.1 Hz), and high-frequency system noise, while preserving the task-related frequency band. Finally, motion artifacts were corrected using the temporal derivative distribution repair (TDDR) [55], ensuring the integrity of the hemodynamic signal.

### Brain activation analysis

Brain activation was analyzed using a general linear model (GLM) for the normal and ICPP group data by gender [56]. The form of the GLM is as follows:

$$\begin{array}{c} \left[\begin{array}{c} y_{1}\\ y_{2}\\ \vdots \\ y_{n} \end{array}\right]=\left[\begin{array}{cccc} 1 & x_{11} & \cdots & x_{1p}\\ 1 & x_{21} & \cdots & x_{2p}\\ \vdots & \vdots & \vdots & \vdots \\ 1 & x_{n1} & \cdots & x_{\mathrm{np}} \end{array}\right]\times \left[\begin{array}{c} \beta_{0}\\ \beta_{1}\\ \vdots \\ \beta_{p} \end{array}\right]+\left[\begin{array}{c} \varepsilon_{1}\\ \varepsilon_{2}\\ \vdots \\ \varepsilon_{n} \end{array}\right] \end{array}$$ (6)

where *y* represents the fNIRS signal observed on a specific channel, *x* is the design matrix, interpreted as the individual hemodynamic responses induced by MA and rest condition in the fNIRS data, *β* denotes the contribution of each explanatory variable to the fNIRS observed signal, ε is the vector of residuals, *n* is the number of data points for each channel, and *p* the number of columns of the design matrix, with *n* > *p* to ensure a valid estimation [57]. The canonical hemodynamic response function (HRF) was selected for the model in this study [58,59]. After data preprocessing, the design matrix for the onset and duration of the MA task and rest phases was input, and the GLM was applied to obtain the channel-wise *β* values. Subsequently, the Shapiro–Wilk test was used to examine the normality of the *β* values, and the Levene test was applied to assess their homogeneity of variance. The results indicated that the *β* values followed a normal distribution or approximated it and exhibited homogeneity of variance (*w* ≈ 1, *P* > 0.05). Then, paired sample *t* test was performed on the *β* values during the MA task and rest phases in the normal and ICPP groups, stratified by gender, to determine which channels were significantly activated during MA [57,60]. Additionally, 2-sample *t* test was conducted on the *β* values for the MA task in the normal and ICPP groups, stratified by gender, to identify channels with significant differences in activation. The significance level was set at false discovery rate (FDR) corrected *P* < 0.05. The normality test of the *β* values was conducted using SPSS 27 software. Data preprocessing, GLM analysis, and statistical analyses were conducted using the NIRS_KIT toolbox [59] on MATLAB r2020b.

### Model building

#### Feature extraction

A total of 344 trial-dependent measurements were obtained during the experiment, with each trial lasting 60 s. In machine learning models, directly inputting all data can lead to the curse of dimensionality, which negatively impacts predictive accuracy. Therefore, extracting relevant features is crucial as it effectively reduces the data dimensionality, improving both the training efficiency and the predictive performance of the model. This study extracted 5 different types of features from the hemoglobin signal, as shown in Fig. 6. Specifically, the feature signals extracted in this study and the rationale behind their selection are presented below:

**Feature A: Time-domain characteristics of the strongest and most frequent occurring negatively correlated channels:** Research has emphasized the importance of negative network strengths in functional connectivity in predicting cognitive and neural performance [41]. Negative correlations in brain activity may reflect specific compensatory mechanisms related to cognitive and neural processes. For each subject, we calculate the Pearson correlation coefficients pairwise across the 10 channels of hemoglobin data per trial, identifying the 2 channels that most frequently exhibit the strongest negative correlation. We then calculate the time-domain characteristics, including the mean, variance, skewness, and kurtosis, for the corresponding channels [61].

**Feature B: The first 3 principal components of each channel using PCA:** PCA is performed on each channel to extract the first 3 principal components, which typically capture the main variance in the data and represent the key information of task-related brain hemodynamics [62,63].

**Feature C: Combining the time-domain characteristics of the strongest and most frequently occurring negatively correlated channels with the first 3 principal components extracted from the corresponding channel using PCA:** The features extracted from A and B are concatenated. Extract the time-domain features and the first 3 principal components from PCA on the 2 channels with the most frequently occurring negative correlation. Data features are extracted from multiple perspectives to capture the basic characteristics and main variation patterns of the relevant channel signals.

**Feature D: The first principal components feature using PCA after empirical mode decomposition (EMD):** EMD is a signal decomposition method that decomposes hemoglobin signals into intrinsic mode functions (IMFs). Subsequently, PCA isolates the single most contributive feature from each IMF. This approach leverages EMD’s adaptability to nonlinear and nonstationary data, along with PCA’s efficiency in capturing the most representative signal dynamics [64].

**Feature E: The activation degree *β* value of each channel obtained by GLM:** GLM quantifies the activation degree in each channel using the *β* value, which represents the strength and direction of task-induced hemodynamic changes in the fNIRS signals. A higher *β* value indicates a stronger task influence on the hemodynamic response, which is crucial for understanding spatial patterns of brain activation [57].

These features integrate time-domain, frequency-domain, and brain functional connectivity features to capture potential patterns of brain activity, thereby ensuring a comprehensive understanding of the hemodynamic response associated with the stimulus.

#### Dataset division and model construction

Five classical machine learning classification algorithms are selected to distinguish between the ICPP and normal groups. These algorithms include 2 supervised learning methods (SVM and KNN), an ensemble learning algorithm (RF), a tree-based model (DT), and a dimensionality reduction method (LDA). These algorithms were selected due to their widespread recognition, inherent interpretability, computational efficiency, and ability to perform effectively even with limited sample sizes. Each algorithm represents a different way of classifying fNIRS data. In binary classification tasks, SVM aims to find a splitting hyperplane that maximizes the margin between data points from different classes [65]. DT works by breaking down complex decision processes into simpler ones, forming a hierarchical tree-like structure. During construction, the DT algorithm strategically selects the best features for splitting. However, this process can lead to overfitting, especially in deep or complex trees [66]. RF improves prediction accuracy and stability by aggregating multiple DTs [67]. LDA effectively classifies data by projecting the data into a lower-dimensional space to maximize between-class separation and minimize within-class variance [68]. KNN determines the class of a sample by identifying the nearest neighbors through distance calculations [69]. All machine learning classification algorithms were implemented using PyCharm 2023.3.5 Community Edition, with the open-source machine learning library scikit-learn 1.2.2 in Python 3.11.7.

The procedure for model building is illustrated in Fig. 6. After the feature extraction process, the dataset was randomly divided into a training set and a testing set with an 8:2 ratio. In the training set, 10-fold cross-validation and grid search were used for hyperparameter optimization, selecting the optimal hyperparameters for each model and making predictions on the test set. Labels were assigned as 1 for the ICPP group and 0 for the normal group. Evaluation measures for the test set, including accuracy, precision, recall, specificity, F1 score, ROC curve, and confusion matrix, were calculated as follows:

$$\begin{array}{c} \begin{array}{c} \text{Accuracy}=\frac{\mathrm{TP}+\mathrm{TN}}{\mathrm{TP}+\mathrm{FP}+\mathrm{FN}+\mathrm{TN}}\times 100\%\\ \text{Precision}=\frac{\mathrm{TP}}{\mathrm{TP}+\mathrm{FP}}\times 100\%\\ \text{Specificity}=\frac{\mathrm{TN}}{\mathrm{TN}+\mathrm{FP}}\times 100\%\\ \text{Recall}=\frac{\mathrm{TP}}{\mathrm{TP}+\mathrm{FN}}\times 100\%\\ F1\;\text{score}=\frac{2\times \left(\text{Precision}\times \text{Recall}\right)}{\text{Precision}+\text{Recall}} \end{array} \end{array}$$ (7)

where TP represents the number of ICPP cases correctly predicted as ICPP, TN indicates the number of normal cases accurately predicted as normal, FP denotes the number of normal cases incorrectly predicted as ICPP, and FN refers to the number of ICPP cases mistakenly predicted as normal. Given the class imbalance in this study, where the normal group is larger than the diseased group, a weighted average strategy was adopted for precision, recall, and F1 score to provide a more objective evaluation of the model’s overall performance. This strategy involves calculating the metric for each class independently and then computing a sum weighted by the number of true instances for each class.

## Data Availability

Data underlying the results presented in this paper are not publicly available at this time but may be obtained from the corresponding authors upon reasonable request and under a licensing agreement.
