# Supplementary material for: Machine Learning-Powered fNIRS Detection of Idiopathic Central Precocious Puberty via Prefrontal Cortex Activation
Source: BME Front. 2026 Mar 25;7:0223. doi: 10.34133/bmef.0223 (PMC13014019; doi:10.34133/bmef.0223)
Supplement: Supplementary 1 — Tables S1 to S5 [file bmef.0223.f1.docx]

**Supplementary Information for Machine Learning-powered fNIRS Detection of Idiopathic Central Precocious Puberty via PFC Activation**

Zeying Li^1^, Lifang Jia^2,3^ Yingxue Zou^2,4^ Mengyu Jia^1^, Limin Zhang^1^, Dongyuan Liu^1^, and Feng Gao^1^

^1^ College of Precision Instruments and Optoelectronics Engineering, Tianjin University, Tianjin 300072, China

^2^ Academy of Medical Engineering and Translational Medicine, Tianjin University, Tianjin 300072, China

^3^ Tianjin Hospital of Tianjin, Tianjin 300211, China

^4^ Children’s Hospital of Tianjin University, Tianjin 300074, China

**Contents:**

**Supplementary Table S1.** Classification performance of different hemoglobin features (gender insensitive)

**Supplementary Table S2.** Classification performance of different hemoglobin features (females)

**Supplementary Table S3.** Classification performance of different hemoglobin features (males)

**Supplementary Table S4.** Summary of correlation analysis between brain activation and demographic data

**Supplementary Table S5.** Correlations between brain activation and clinical data in the ICPP group

**Supplementary Table S1** Classification performance of different hemoglobin features (gender insensitive)

| Feature | Hemoglobin | Algorithm | Accuracy | Precision | Recall | F1-Score | Specificity |
| --- | --- | --- | --- | --- | --- | --- | --- |
|  | HbO | LDA | 83.58 | 83.62 | 83.58 | 83.58 | 82.35 |
|  |  | SVM | 82.09 | 82.58 | 82.09 | 82.04 | 76.47 |
|  |  | DT | **86.57** | **83.61** | **86.57** | **86.57** | **85.29** |
|  |  | RF | 86.57 | 87.43 | 86.57 | 86.51 | 79.41 |
|  |  | KNN | 77.61 | 77.86 | 77.61 | 77.58 | 73.53 |
|  | HbR | LDA | 58.21 | 58.20 | 58.21 | 58.15 | 61.76 |
| (a) |  | SVM | 74.63 | 74.66 | 74.63 | 74.63 | 73.53 |
|  |  | DT | 62.69 | 62.68 | 62.69 | 62.67 | 64.71 |
|  |  | RF | 70.15 | 70.25 | 70.15 | 70.14 | 67.65 |
|  |  | KNN | 73.13 | 73.24 | 73.13 | 73.12 | 70.59 |
|  | HbT | LDA | 68.66 | 68.69 | 68.66 | 68.66 | 67.65 |
|  |  | SVM | 61.19 | 61.67 | 61.19 | 60.93 | 52.94 |
|  |  | DT | 53.73 | 53.93 | 53.73 | 52.33 | 70.59 |
|  |  | RF | 70.15 | 70.15 | 70.15 | 70.15 | 70.59 |
|  |  | KNN | 62.69 | 62.89 | 62.69 | 62.44 | 70.59 |
|  | HbO | LDA | 52.24 | 52.24 | 52.24 | 52.24 | 52.94 |
|  |  | SVM | 61.19 | 61.29 | 61.19 | 61.02 | 67.65 |
|  |  | DT | 47.76 | 46.00 | 47.76 | 42.91 | 76.47 |
|  |  | RF | **64.18** | **64.20** | **64.18** | **64.13** | **67.65** |
|  |  | KNN | 53.73 | 53.93 | 53.73 | 52.33 | 70.59 |
|  | HbR | LDA | 56.72 | 56.70 | 56.72 | 56.70 | 58.82 |
| (b) |  | SVM | 55.22 | 55.37 | 55.22 | 54.49 | 67.65 |
|  |  | DT | 53.73 | 53.71 | 53.73 | 53.42 | 61.76 |
|  |  | RF | 58.21 | 58.20 | 58.21 | 58.15 | 61.76 |
|  |  | KNN | 56.72 | 56.77 | 56.72 | 56.42 | 64.71 |
|  | HbT | LDA | 44.78 | 44.53 | 44.78 | 44.40 | 52.94 |
|  |  | SVM | 46.27 | 45.63 | 46.27 | 44.92 | 61.76 |
|  |  | DT | 47.76 | 45.43 | 47.76 | 41.74 | 79.41 |
|  |  | RF | 53.73 | 54.29 | 53.73 | 51.11 | 76.47 |
|  |  | KNN | 62.69 | 65.61 | 62.69 | 60.57 | 85.29 |
|  | HbO | LDA | 83.58 | 83.87 | 83.58 | 83.56 | 79.41 |
|  |  | SVM | 83.58 | 83.62 | 83.58 | 83.58 | 82.35 |
|  |  | DT | **86.57** | **86.61** | **86.57** | **86.57** | **85.29** |
|  |  | RF | 83.58 | 83.87 | 83.58 | 83.56 | 79.41 |
|  |  | KNN | 76.12 | 76.46 | 76.12 | 76.01 | 82.35 |
|  | HbR | LDA | 65.67 | 65.67 | 65.67 | 65.66 | 67.65 |
| (c) |  | SVM | 64.18 | 64.26 | 64.18 | 64.16 | 61.76 |
|  |  | DT | 61.19 | 61.73 | 61.19 | 60.56 | 73.53 |
|  |  | RF | 79.10 | 80.02 | 79.10 | 78.91 | 88.24 |
|  |  | KNN | 62.69 | 62.71 | 62.69 | 62.69 | 61.76 |
|  | HbT | LDA | 59.70 | 59.83 | 59.70 | 59.43 | 67.65 |
|  |  | SVM | 53.73 | 53.75 | 53.73 | 53.15 | 64.71 |
|  |  | DT | 52.24 | 75.40 | 52.24 | 37.40 | 100.00 |
|  |  | RF | 71.64 | 71.78 | 71.64 | 71.57 | 76.47 |
|  |  | KNN | 52.69 | 64.03 | 62.69 | 61.55 | 79.41 |
|  | HbO | LDA | 52.24 | 52.24 | 52.24 | 52.24 | 52.94 |
|  |  | SVM | 50.75 | 25.75 | 50.75 | 34.17 | 100.00 |
|  |  | DT | **59.70** | **59.83** | **59.70** | **59.43** | **67.65** |
|  |  | RF | 58.21 | 58.37 | 58.21 | 57.81 | 67.65 |
|  |  | KNN | 50.75 | 50.69 | 50.75 | 50.61 | 55.88 |
|  | HbR | LDA | 52.24 | 52.20 | 52.24 | 52.17 | 55.88 |
| (d) |  | SVM | 56.72 | 56.70 | 56.72 | 56.70 | 58.82 |
|  |  | DT | 44.78 | 32.33 | 44.78 | 33.51 | 85.29 |
|  |  | RF | 52.24 | 52.19 | 52.24 | 51.46 | 64.71 |
|  |  | KNN | 56.72 | 56.88 | 56.72 | 56.17 | 67.65 |
|  | HbT | LDA | 46.27 | 46.27 | 46.27 | 46.27 | 47.06 |
|  |  | SVM | 52.24 | 52.35 | 52.24 | 49.89 | 73.53 |
|  |  | DT | 59.70 | 59.73 | 59.70 | 59.70 | 58.82 |
|  |  | RF | 52.24 | 52.46 | 52.24 | 49.15 | 76.47 |
|  |  | KNN | 50.75 | 50.45 | 50.75 | 43.80 | 85.29 |
|  | HbO | LDA | 55.22 | 55.72 | 55.22 | 53.61 | 73.53 |
|  |  | SVM | 64.18 | 65.40 | 64.18 | 63.28 | 79.41 |
|  |  | DT | **65.67** | **65.67** | **65.67** | **65.66** | **67.65** |
|  |  | RF | 59.70 | 59.73 | 59.70 | 59.70 | 58.82 |
|  |  | KNN | 55.22 | 55.71 | 55.22 | 54.66 | 44.12 |
|  | HbR | LDA | 59.70 | 60.29 | 59.70 | 58.88 | 73.53 |
| (e) |  | SVM | 56.72 | 58.06 | 56.72 | 64.26 | 79.41 |
|  |  | DT | 59.70 | 65.54 | 59.70 | 55.05 | 91.18 |
|  |  | RF | 58.21 | 58.20 | 58.21 | 58.15 | 61.76 |
|  |  | KNN | 46.27 | 45.63 | 46.27 | 44.92 | 61.76 |
|  | HbT | LDA | 52.24 | 52.24 | 52.24 | 52.24 | 52.94 |
|  |  | SVM | 49.25 | 48.36 | 49.25 | 45.05 | 76.47 |
|  |  | DT | 53.73 | 54.08 | 53.73 | 51.77 | 73.53 |
|  |  | RF | 64.18 | 66.95 | 64.18 | 62.41 | 85.29 |
|  |  | KNN | 59.70 | 61.17 | 59.70 | 57.99 | 79.41 |

HbO: Oxy-hemoglobin; HbR: Deoxy-hemoglobin; HbT: Total-hemoglobin; LDA: Linear discriminant analysis; SVM: Support vector machine; DT: Decision tree; RF: Random forest; KNN: K-nearest neighbors; (a): Time-domain characteristics of the strongest and most frequent occurring negatively correlated channels; (b): The first three principal components of each channel using PCA; (c): Combining the time-domain characteristics of the strongest and most frequently occurring negatively correlated channels with the first three principal components extracted from corresponding channel using PCA; (d): The first principal components feature using PCA after EMD; (e): The activation degree β value of each channel obtained by GLM. Bold font indicates the best classifier performance by the corresponding feature set.

**Supplementary Table S2.** Classification performance of different hemoglobin features (females)

| Feature | Hemoglobin | Algorithm | Accuracy | Precision | Recall | F1-Score | Specificity |
| --- | --- | --- | --- | --- | --- | --- | --- |
|  | HbO | LDA | 84.44 | 85.80 | 84.44 | 83.95 | 68.42 |
|  |  | SVM | 82.22 | 82.81 | 82.22 | 81.81 | 68.42 |
|  |  | DT | 82.22 | 84.11 | 82.22 | 81.48 | 63.16 |
|  |  | RF | 86.67 | 86.67 | 86.67 | 86.67 | 84.21 |
|  |  | KNN | 84.44 | 84.76 | 84.44 | 84.20 | 73.68 |
|  | HbR | LDA | 66.67 | 67.65 | 66.67 | 66.87 | 68.42 |
| (a) |  | SVM | 82.22 | 82.72 | 82.22 | 82.31 | 84.21 |
|  |  | DT | 75.56 | 75.43 | 75.56 | 75.46 | 68.42 |
|  |  | RF | 75.56 | 77.67 | 75.56 | 75.68 | 84.21 |
|  |  | KNN | 80.00 | 79.92 | 80.00 | 79.92 | 73.68 |
|  | HbT | LDA | 60.00 | 60.00 | 60.00 | 60.00 | 52.63 |
|  |  | SVM | 64.44 | 64.04 | 64.44 | 64.12 | 52.63 |
|  |  | DT | 62.22 | 64.14 | 62.22 | 62.41 | 68.42 |
|  |  | RF | 51.11 | 53.24 | 51.11 | 51.26 | 57.89 |
|  |  | KNN | 77.78 | 77.78 | 77.78 | 77.78 | 73.68 |
|  | HbO | LDA | 46.67 | 49.97 | 46.67 | 45.87 | 63.16 |
|  |  | SVM | 55.56 | 58.85 | 55.56 | 55.38 | 68.42 |
|  |  | DT | 51.11 | 51.11 | 51.11 | 51.11 | 42.11 |
|  |  | RF | 48.89 | 49.93 | 48.89 | 49.19 | 47.37 |
|  |  | KNN | 53.33 | 55.98 | 53.33 | 53.33 | 63.16 |
|  | HbR | LDA | 51.11 | 57.71 | 51.11 | 48.69 | 78.95 |
| (b) |  | SVM | 57.78 | 58.09 | 57.78 | 57.91 | 52.63 |
|  |  | DT | 48.89 | 65.22 | 48.89 | 40.67 | 94.74 |
|  |  | RF | 46.67 | 49.30 | 46.67 | 46.46 | 57.89 |
|  |  | KNN | 48.89 | 51.31 | 48.89 | 48.89 | 57.89 |
|  | HbT | LDA | 55.56 | 58.85 | 55.56 | 55.38 | 68.42 |
|  |  | SVM | 57.78 | 61.90 | 57.78 | 57.40 | 73.68 |
|  |  | DT | 57.78 | 61.90 | 57.78 | 57.40 | 73.68 |
|  |  | RF | 55.56 | 58.85 | 55.56 | 55.38 | 68.42 |
|  |  | KNN | 48.89 | 65.22 | 48.89 | 40.67 | 94.74 |
|  | HbO | LDA | 84.44 | 85.80 | 84.44 | 83.95 | 68.42 |
|  |  | SVM | 80.00 | 80.13 | 80.00 | 79.69 | 68.42 |
|  |  | DT | 82.22 | 84.11 | 82.22 | 81.48 | 63.16 |
|  |  | RF | **91.11** | **91.11** | **91.11** | **91.11** | **89.47** |
|  |  | KNN | 82.22 | 82.22 | 82.22 | 82.22 | 78.95 |
|  | HbR | LDA | 68.89 | 69.35 | 68.89 | 68.98 | 68.00 |
| (c) |  | SVM | 73.33 | 73.33 | 73.33 | 73.33 | 76.00 |
|  |  | DT | 84.44 | 84.59 | 84.44 | 84.48 | 84.00 |
|  |  | RF | 80.00 | 80.83 | 80.00 | 80.06 | 76.00 |
|  |  | KNN | 75.56 | 75.48 | 75.56 | 75.48 | 80.00 |
|  | HbT | LDA | 75.56 | 75.78 | 75.56 | 75.63 | 73.68 |
|  |  | SVM | 62.22 | 66.80 | 62.22 | 61.89 | 78.95 |
|  |  | DT | 57.78 | 68.38 | 57.78 | 55.19 | 89.47 |
|  |  | RF | 68.89 | 69.47 | 68.89 | 69.04 | 68.42 |
|  |  | KNN | 60.00 | 62.40 | 60.00 | 60.12 | 68.42 |
|  | HbO | LDA | 60.00 | 76.36 | 60.00 | 47.14 | 5.26 |
|  |  | SVM | 57.78 | 58.09 | 57.78 | 57.91 | 52.63 |
|  |  | DT | 53.33 | 55.12 | 53.33 | 53.56 | 57.89 |
|  |  | RF | 53.33 | 55.12 | 53.33 | 53.56 | 57.89 |
|  |  | KNN | 53.33 | 56.99 | 53.33 | 52.92 | 68.42 |
|  | HbR | LDA | 62.22 | 66.80 | 62.22 | 61.89 | 78.95 |
| (d) |  | SVM | 55.56 | 60.11 | 55.56 | 54.89 | 73.68 |
|  |  | DT | 48.89 | 60.16 | 48.89 | 42.69 | 89.47 |
|  |  | RF | 64.44 | 65.88 | 64.44 | 64.66 | 68.42 |
|  |  | KNN | 55.56 | 66.73 | 55.56 | 52.26 | 89.47 |
|  | HbT | LDA | 53.33 | 56.99 | 53.33 | 52.92 | 68.42 |
|  |  | SVM | 44.44 | 76.01 | 44.44 | 29.75 | 100.00 |
|  |  | DT | 46.67 | 52.76 | 46.67 | 42.72 | 78.95 |
|  |  | RF | 51.11 | 55.04 | 51.11 | 50.38 | 68.42 |
|  |  | KNN | 51.11 | 67.64 | 51.11 | 44.26 | 94.74 |
|  | HbO | LDA | 57.78 | 63.48 | 57.78 | 56.85 | 78.95 |
|  |  | SVM | 62.22 | 68.74 | 62.22 | 61.39 | 84.21 |
|  |  | DT | 60.00 | 61.42 | 60.00 | 60.24 | 63.16 |
|  |  | RF | 68.89 | 73.17 | 68.89 | 68.77 | 84.21 |
|  |  | KNN | 48.89 | 51.31 | 48.89 | 48.89 | 57.89 |
|  | HbR | LDA | 55.56 | 56.96 | 55.56 | 55.82 | 57.89 |
| (e) |  | SVM | 42.22 | 17.83 | 42.22 | 25.07 | 100.00 |
|  |  | DT | 42.22 | 17.83 | 42.22 | 25.07 | 100.00 |
|  |  | RF | 60.00 | 69.90 | 60.00 | 58.02 | 89.47 |
|  |  | KNN | 48.89 | 54.03 | 48.89 | 46.88 | 73.68 |
|  | HbT | LDA | 46.67 | 49.30 | 46.67 | 46.46 | 57.89 |
|  |  | SVM | 46.67 | 61.87 | 46.67 | 36.89 | 94.74 |
|  |  | DT | 40.00 | 41.43 | 40.00 | 32.73 | 78.95 |
|  |  | RF | 46.67 | 76.43 | 46.67 | 34.13 | 100.00 |
|  |  | KNN | 51.11 | 54.07 | 51.11 | 50.92 | 63.16 |

HbO: Oxy-hemoglobin; HbR: Deoxy-hemoglobin; HbT: Total-hemoglobin; LDA: Linear discriminant analysis; SVM: Support vector machine; DT: Decision tree; RF: Random forest; KNN: K-nearest neighbors; (a): Time-domain characteristics of the strongest and most frequent occurring negatively correlated channels; (b): The first three principal components of each channel using PCA; (c): Combining the time-domain characteristics of the strongest and most frequently occurring negatively correlated channels with the first three principal components extracted from corresponding channel using PCA; (d): The first principal components feature using PCA after EMD; (e): The activation degree β value of each channel obtained by GLM. Bold font indicates the best classifier performance by the best feature set.

**Supplementary Table S3.** Classification performance of different hemoglobin features (males)

| Feature | Hemoglobin | Algorithm | Accuracy | Precision | Recall | F1-Score | Specificity |
| --- | --- | --- | --- | --- | --- | --- | --- |
|  | HbO | LDA | **91.30** | **91.30** | **91.30** | **91.30** | **90.91** |
|  |  | SVM | 86.96 | 87.29 | 86.96 | 86.96 | 90.91 |
|  |  | DT | 73.91 | 74.47 | 73.91 | 73.61 | 63.64 |
|  |  | RF | **91.30** | **91.30** | **91.30** | **91.30** | **90.91** |
|  |  | KNN | 82.61 | 83.65 | 82.61 | 82.54 | 90.91 |
|  | HbR | LDA | 60.87 | 61.10 | 60.87 | 60.87 | 63.64 |
| (a) |  | SVM | 78.26 | 78.56 | 78.26 | 78.26 | 81.82 |
|  |  | DT | 60.87 | 61.10 | 60.87 | 60.87 | 63.64 |
|  |  | RF | 73.91 | 74.85 | 73.91 | 73.81 | 81.82 |
|  |  | KNN | 69.57 | 69.83 | 69.57 | 69.57 | 72.73 |
|  | HbT | LDA | 69.57 | 69.83 | 69.57 | 69.57 | 72.73 |
|  |  | SVM | 65.22 | 66.77 | 65.22 | 63.84 | 45.45 |
|  |  | DT | 52.17 | 51.53 | 52.17 | 49.26 | 27.27 |
|  |  | RF | 52.17 | 52.01 | 52.17 | 51.99 | 45.45 |
|  |  | KNN | 65.22 | 65.42 | 65.22 | 64.82 | 54.55 |
|  | HbO | LDA | 60.87 | 60.80 | 60.87 | 60.72 | 54.55 |
|  |  | SVM | 47.83 | 48.16 | 47.83 | 47.63 | 54.55 |
|  |  | DT | 65.22 | 65.22 | 65.22 | 65.22 | 63.64 |
|  |  | RF | 56.52 | 56.52 | 56.52 | 56.52 | 54.55 |
|  |  | KNN | 65.22 | 79.86 | 65.22 | 61.16 | 100.00 |
|  | HbR | LDA | 52.17 | 52.37 | 52.17 | 52.17 | 54.55 |
| (b) |  | SVM | 56.52 | 58.12 | 56.52 | 55.52 | 72.73 |
|  |  | DT | 47.83 | 48.16 | 47.83 | 47.63 | 54.55 |
|  |  | RF | 47.83 | 48.59 | 47.83 | 44.72 | 72.73 |
|  |  | KNN | 39.13 | 37.08 | 39.13 | 35.51 | 63.64 |
|  | HbT | LDA | 52.17 | 52.37 | 52.17 | 52.17 | 54.55 |
|  |  | SVM | 56.52 | 58.12 | 56.52 | 55.52 | 72.73 |
|  |  | DT | 47.83 | 48.16 | 47.83 | 47.63 | 54.55 |
|  |  | RF | 47.83 | 48.59 | 47.83 | 44.72 | 72.73 |
|  |  | KNN | 39.13 | 37.08 | 39.13 | 35.51 | 63.64 |
|  | HbO | LDA | **91.30** | **91.30** | **91.30** | **91.30** | **90.91** |
|  |  | SVM | **91.30** | **91.30** | **91.30** | **91.30** | **90.91** |
|  |  | DT | 82.61 | 82.61 | 82.61 | 82.61 | 81.82 |
|  |  | RF | **91.30** | **91.30** | **91.30** | **91.30** | **90.91** |
|  |  | KNN | 82.61 | 87.25 | 82.61 | 82.21 | 100.00 |
|  | HbR | LDA | 60.87 | 61.20 | 60.87 | 59.95 | 45.45 |
| (c) |  | SVM | 60.87 | 61.10 | 60.87 | 60.87 | 63.44 |
|  |  | DT | 56.52 | 56.52 | 56.52 | 56.52 | 54.55 |
|  |  | RF | 73.91 | 73.91 | 73.91 | 73.91 | 72.73 |
|  |  | KNN | 47.83 | 48.59 | 47.83 | 44.72 | 72.73 |
|  | HbT | LDA | 69.57 | 71.33 | 69.57 | 69.22 | 81.82 |
|  |  | SVM | 60.87 | 60.80 | 60.87 | 60.72 | 54.55 |
|  |  | DT | 47.83 | 48.86 | 47.83 | 37.34 | 90.91 |
|  |  | RF | 69.57 | 74.61 | 69.57 | 68.38 | 90.91 |
|  |  | KNN | 34.78 | 34.91 | 34.78 | 34.78 | 36.36 |
|  | HbO | LDA | 60.87 | 60.80 | 60.87 | 60.72 | 54.55 |
|  |  | SVM | 52.17 | 58.70 | 52.17 | 44.77 | 90.91 |
|  |  | DT | 52.17 | 52.90 | 52.17 | 51.63 | 63.64 |
|  |  | RF | 73.91 | 74.85 | 73.91 | 73.81 | 81.82 |
|  |  | KNN | 43.48 | 43.28 | 43.48 | 41.27 | 63.44 |
|  | HbR | LDA | 43.48 | 42.13 | 43.48 | 38.66 | 72.73 |
| (d) |  | SVM | 52.17 | 53.73 | 52.17 | 50.31 | 72.73 |
|  |  | DT | 56.52 | 76.28 | 56.52 | 44.80 | 9.09 |
|  |  | RF | 39.13 | 38.70 | 39.13 | 37.73 | 54.55 |
|  |  | KNN | 52.17 | 52.37 | 52.17 | 52.17 | 54.55 |
|  | HbT | LDA | 47.83 | 48.16 | 47.83 | 47.63 | 54.55 |
|  |  | SVM | 43.48 | 42.13 | 43.48 | 38.66 | 72.73 |
|  |  | DT | 60.87 | 62.11 | 60.87 | 60.42 | 72.73 |
|  |  | RF | 43.48 | 43.28 | 43.48 | 41.27 | 63.64 |
|  |  | KNN | 69.57 | 81.40 | 69.57 | 66.97 | 100.00 |
|  | HbO | LDA | 47.83 | 48.16 | 47.83 | 47.63 | 54.55 |
|  |  | SVM | 60.87 | 62.11 | 60.87 | 60.42 | 72.73 |
|  |  | DT | 60.87 | 61.10 | 60.87 | 60.87 | 63.44 |
|  |  | RF | 52.17 | 52.90 | 52.17 | 51.63 | 63.44 |
|  |  | KNN | 65.22 | 71.61 | 65.22 | 63.15 | 90.91 |
|  | HbR | LDA | 60.87 | 64.17 | 60.87 | 59.34 | 81.82 |
| (e) |  | SVM | 60.87 | 68.31 | 60.87 | 57.54 | 90.91 |
|  |  | DT | 65.22 | 67.83 | 65.22 | 64.41 | 81.82 |
|  |  | RF | 60.87 | 62.11 | 60.87 | 60.42 | 72.73 |
|  |  | KNN | 78.26 | 78.39 | 78.26 | 78.18 | 72.73 |
|  | HbT | LDA | 65.22 | 67.83 | 65.22 | 64.41 | 81.82 |
|  |  | SVM | 65.22 | 65.95 | 65.22 | 65.09 | 72.73 |
|  |  | DT | 60.87 | 62.57 | 60.87 | 58.49 | 36.36 |
|  |  | RF | 65.22 | 65.42 | 65.22 | 64.82 | 54.55 |
|  |  | KNN | 65.22 | 65.95 | 65.22 | 65.09 | 72.73 |

HbO: Oxy-hemoglobin; HbR: Deoxy-hemoglobin; HbT: Total-hemoglobin; LDA: Linear discriminant analysis; SVM: Support vector machine; DT: Decision tree; RF: Random forest; KNN: K-nearest neighbors; (a): Time-domain characteristics of the strongest and most frequent occurring negatively correlated channels; (b): The first three principal components of each channel using PCA; (c): Combining the time-domain characteristics of the strongest and most frequently occurring negatively correlated channels with the first three principal components extracted from corresponding channel using PCA; (d): The first principal components feature using PCA after EMD; (e): The activation degree β value of each channel obtained by GLM. Bold font indicates the best classifier performance by the best feature set.

**Supplementary Table S4.** Summary of correlation analysis between brain activation and demographic data

|  |  |  |  | Age | | Education | |
| --- | --- | --- | --- | --- | --- | --- | --- |
|  |  | Gender | Channel | r | p-value | r | p-value |
|  | HbO | Female | 5 | -0.043 | 0.75 | -0.043 | 0.75 |
| Normal |  | Male | 8 | 0.246 | 0.207 | 0.243 | 0.212 |
|  | HbR | Female | 5 | -0.004 | 0.974 | -0.004 | 0.974 |
|  |  | Male | 4 | 0.282 | 0.146 | 0.271 | 0.163 |
|  | HbO | Female | 5 | -0.037 | 0.793 | -0.059 | 0.671 |
| ICPP |  | Male | 8 | 0.051 | 0.797 | 0.051 | 0.797 |
|  | HbR | Female | 5 | -0.005 | 0.972 | -0.028 | 0.842 |
|  |  | Male | 4 | -0.143 | 0.467 | -0.143 | 0.467 |

**Supplementary Table S5.** Correlations between brain activation and clinical data in the ICPP group

|  |  |  | Bone age | | LH | | FSH | | LH/FSH | | Estradiol | | Testosterone | |
| --- | --- | --- | --- | --- | --- | --- | --- | --- | --- | --- | --- | --- | --- | --- |
|  | Gender | Channel | r | p-value | r | p-value | r | p-value | r | p-value | r | p-value | r | p-value |
| HbO | Female | 5 | 0.084 | 0.646 | 0.313 | 0.071 | 0.360 | 0.029 | 0.292 | 0.088 | 0.307 | 0.154 | /* | /* |
|  | Male | 8 | 0.388 | 0.170 | 0.193 | 0.528 | -0.234 | 0.464 | 0.466 | 0.127 | /* | /* | 0.220 | 0.515 |
| HbR | Female | 5 | 0.173 | 0.353 | 0.273 | 0.119 | 0.312 | 0.06 | 0.311 | 0.069 | 0.229 | 0.293 | /* | /* |
|  | Male | 4 | 0.328 | 0.253 | -0.271 | 0.371 | -0.571 | 0.053 | 0.258 | 0.418 | /* | /* | -0.128 | 0.708 |

**Females are mainly concerned about the level of estradiol, while males are mainly concerned about the level of testosterone.*
